# Limited Ability to Adjust N2 Amplitude During Dual Task Walking in People With Drug-Resistant Juvenile Myoclonic Epilepsy

**DOI:** 10.3389/fneur.2022.793212

**Published:** 2022-02-07

**Authors:** Mor Yam, Sigal Glatt, Shai Nosatzki, Anat Mirelman, Jeffrey M. Hausdorff, Lilach Goldstein, Nir Giladi, Firas Fahoum, Inbal Maidan

**Affiliations:** ^1^Laboratory of Early Markers of Neurodegeneration, Centre for the Study of Movement, Cognition, and Mobility, Tel Aviv Sourasky Medical Centre, Neurological Institute, Tel Aviv, Israel; ^2^Sagol School of Neuroscience, Tel Aviv University, Tel Aviv, Israel; ^3^Department of Neurology and Neurosurgery, Sackler Faculty of Medicine, Tel Aviv University, Tel Aviv, Israel; ^4^Department of Physical Therapy, Sackler Faculty of Medicine, Tel Aviv University, Tel Aviv, Israel; ^5^Rush Alzheimer's Disease Center and Department of Orthopaedic Surgery, Rush University Medical Center, Chicago, IL, United States; ^6^Epilepsy Unit, Tel Aviv Sourasky Medical Centre, Neurological Institute, Tel Aviv, Israel

**Keywords:** epilepsy, event-related potentials (ERP), dual-task (DT), drug-resistant, juvenile myoclonic epilepsy (JME)

## Abstract

Juvenile myoclonic epilepsy (JME) is one of the most common epileptic syndromes; it is estimated to affect 1 in 1,000 people worldwide. Most people with JME respond well to medication, but up to 30% of them are drug-resistant. To date, there are no biomarkers for drug resistance in JME, and the poor response to medications is identified in retrospect. People with JME have frontal dysfunction manifested as impaired attention and difficulties in inhibiting habitual responses and these dysfunctions are more pronounced in drug-resistant individuals. Frontal networks play an important role in walking and therefore, gait can be used to overload the neural system and expose subtle changes between people with drug-responsive and drug-resistant JME. Electroencephalogram (EEG) is a promising tool to explore neural changes during real-time functions that combine a cognitive task while walking (dual tasking, DT). This exploratory study aimed to examine the alteration in electrical brain activity during DT in people with drug-responsive and drug-resistant JME. A total of 32 subjects (14 males and 18 females) participated: 11 drug-responsive (ages: 31.50 ± 1.50) and 8 drug-resistant (27.27 ± 2.30) people with JME, and 13 healthy controls (29.46 ± 0.69). The participants underwent EEG examination during the performance of the visual Go/NoGo (vGNG) task while sitting and while walking on a treadmill. We measured latencies and amplitudes of N2 and P3 event-related potentials, and the cognitive performance was assessed by accuracy rate and response time of Go/NoGo events. The results demonstrated that healthy controls had earlier N2 and P3 latencies than both JME groups (N2: *p* = 0.034 and P3: *p* = 0.011), however, a limited ability to adjust the N2 amplitude during walking was noticeable in the drug-resistant compared to drug-responsive. The two JME groups had lower success rates (drug-responsive *p* < 0.001, drug-resistant *p* = 0.004) than healthy controls, but the drug-resistant showed longer reaction times compared to both healthy controls (*p* = 0.033) and drug-responsive (*p* = 0.013). This study provides the first evidence that people with drug-resistant JME have changes in brain activity during highly demanding tasks that combine cognitive and motor functions compared to people with drug-responsive JME. Further research is needed to determine whether these alterations can be used as biomarkers to drug response in JME.

## Introduction

Epilepsy is amongst the most common chronic neurological disorders affecting more than 70 million people worldwide (1). Juvenile myoclonic epilepsy (JME) is a generalized epilepsy syndrome, accounting for up to 10% of all epilepsies (2). It is characterized by several generalized seizure types and diffuse epileptiform activity that is maximal over frontocentral regions (3). People with JME usually respond well to antiseizure medications (ASMs), yet about 15–30% of them will continue to experience seizures despite appropriate ASMs and suffer from drug-resistant epilepsy (DRE) (4–6). It is well known that frontal dysfunction characterized people with JME however, it is exacerbated in people with drug-resistant JME (5, 7–9). To date, despite several attempts to find a marker for DRE (10–12), there are no biomarkers for drug resistance in people with JME, and the poor response to medications is identified retrospectively.

Two frontal cognitive functions that are impaired in people with JME are attention and inhibitory control (13). The visual Go/NoGo (vGNG) task (14) is a classic cognitive task that examines these aspects of attention and response delay (15). In addition, these cognitive functions have been related to gait, especially by using the dual-task (DT) paradigm that combines walking and a cognitive task (16, 17). Engaging in an attention-demanding task while walking requires cognitive resources that can become overburdened and impair motor and cognitive performance (17). Dual-task walking has been widely examined in different neurodegenerative diseases (18) but has not yet been tested in people with epilepsy. Therefore, performing the vGNG task while walking, a complex task that largely relies on frontal functions, may reveal subtle frontal changes that are sensitive to differentiate between people with drug-responsive and drug-resistant JME.

Evaluating event-related potentials (ERPs) by electroencephalogram (EEG) during the vGNG task results in a negative potential at 200–400 ms following the event (N2) and a positive potential at 300–550 ms following the event (P3) (19–21). Both N2 and P3 components are particularly prominent in anterior or frontocentral scalp sites (22). In addition, both ERPs have been linked to attention and response inhibition processes [(22, 23)] which are specific cognitive domains known to be impaired in JME (13). Several studies revealed alterations in both the N2 and P3 components in people with epilepsy. Most studies found prolonged latencies and reduced amplitudes of both components in people with epilepsy, compared to healthy controls (24–27). However, none of these studies included people with JME and almost none of them referred to the drug-responsiveness status of the patient. One study [(27) did refer to the drug-responsiveness status, but not according to the formal International League against Epilepsy [ILAE] definition of drug-resistant. Therefore, this exploratory study aimed to examine the differences in ERPs measures during vGNG tasks performed during sitting and walking between people with drug-resistant JME, people with drug-responsive JME, and healthy controls. We hypothesized that the changes in ERPs during simple and highly demanding motor-cognitive tasks can be used as an additional tool to distinguish people with drug-resistant JME from people with drug-responsive JME and in addition, it might reveal new insights regarding the neural mechanisms that distinguish these two groups of JME.

## Materials and Methods

### Participants

A total of 32 subjects, 19 people with JME (11 drug-responsive and 8 drug-resistant) and 13 healthy controls participated in this study. People with JME were recruited from the Epilepsy and EEG unit, of the Neurological Institute at Tel Aviv Sourasky Medical Center. People with epilepsy were included if they had a JME diagnosis and were at least 18 years old. Drug responsiveness status (responsive or resistant) was determined according to the ILAE' definition of drug-resistant epilepsy as “failure of adequate trials of two tolerated, appropriately chosen and used antiseizures medications (ASMs) schedules (whether as monotherapies or in combination) to achieve sustained seizure freedom” (28). People with epilepsy were tested on their regular medications, to assess performance in their usual clinical state. Age and gender-matched controls without any neurological or psychiatric disorder were included. The study was approved by the local ethical committee according to the principles of the Declaration of Helsinki. All participants gave their informed written consent before participation.

### Procedures

All eligible participants underwent a clinical evaluation and an EEG recording. The EEG examination included the vGNG (14) task that was performed while seated [sitting single task, sitting ST] and while walking on a treadmill (DT). The EEG assessment also included 4 min of resting state while sitting with eyes closed and 4 min of simple walking on the treadmill while looking straight ahead with eyes open. The treadmill walking speed was set according to the comfortable speed of the subject. Subjects were asked to place their hands on the treadmill rail throughout the test to minimize movement artifacts. After the EEG recording was complete, the participants underwent cognitive and clinical assessments. The cognitive assessment included the Montreal Cognitive Assessment (MoCA) which provides a global cognitive function assessment (29), and the color trail test (CTT), which evaluates visual scanning, attention, inhibition, and cognitive flexibility (30). The clinical assessment included a (a) questionnaire of personal characteristic (age, gender, height and weight, marital status, number of years of education, etc.), (b) disease characterization for the people with JME (frequency and type of seizures and medication regimen), and (c) quality of life questionnaire [world health organization quality of life, see (31, 32)].

### Visual Go-NoGo Task

The vGNG task contains two types of stimuli to which the subjects are exposed and to which they are required to react. The different cues are the Go cue, in which the subjects are requested to respond as quickly and as accurately as possible by pressing a keypad, and the NoGo cue where the subjects are requested to inhibit their response and not click the keypad. In this study, the Go cue stimuli were English alphabetic letters (“A,” “B,” “C,” “D,” etc.) and the NoGo cue stimuli was the letter “X.” Eighty percent of the cues were Go cues (320 Go cues), and the rest 20% were NoGo cues (80 NoGo cues), which were distributed randomly along with the experiment. Each condition (Sit/Walk) included two sessions; each session comprised 200 trials that lasted 6 min. Between the two sessions, there was a 1-min intersession interval. The performance in the vGNG task was assessed by the correct percent of Go trials, correct percent of NoGo trials, the total number of correct responses, and average response time [in milliseconds (ms)] to the Go trials.

### EEG Acquisition and Preprocessing

The EEG was recorded *via* the EGI system with 64-channels (EGI GES400). Electrooculogram (EOG) was recorded using 4 channels, 2 channels placed above and below each eye. The EEG reference electrodes were positioned bilaterally on the mastoid bones behind the ears. The EEG data were preprocessed using the EEGLAB open-source MATLAB software package (33). The preprocessing included a bandpass filter with a finite impulse response filter of 0.5–40 Hz, to discard the low band (e.g., baseline drift and motion artifacts) and high band [e.g., electromyogram (EMG)] artifacts. Channels with prominent artifacts were removed based on visual inspection. Next, the data were referenced to the average of all scalp electrodes, and independent component analysis was performed on the dataset to remove EOG and EMG artifacts (34). The average number of removed independent components related to eye blinks and muscle artifacts was 10.8 (±7.7) across subjects.

Finally, for each of the four trial types (“Sit-Go,” “Sit-NoGo,” “Walk-Go,” and “Walk-NoGo”), the signal was divided into 1250 ms epochs (trials) of 250 ms pre-event and 1000 ms postevent. The 250 ms seconds pre-event were used as a baseline for the 1000 ms postevent. Go epochs trials (“Sit-Go” and “Walk-Go”) for which no press button occurred, a press button occurred too early (under 100 ms poststimulus) or too late (above 425 ms poststimulus) were discarded from further analysis. NoGo trials (“Sit-NoGo” and “Walk-NoGo”), for which a press button occurred were also excluded from the dataset. Finally, all the epochs were tested for the presence of residual noise. We calculated the SD amplitude for each electrode and removed epochs with amplitudes exceeding 5 SD. For the Go epochs, an average of 259.2 (±47.1) epochs were obtained while sitting, and 233.5 (±67.4) while walking (maximum number of Go epochs was 320) across subjects. For the NoGo epochs, an average of 56.6 (±9.2) epochs were obtained while sitting, and 50.2 (±15.2) while walking (maximum number of Go epochs was 80) across subjects.

It is important to note that there was no overlap between the ERPs of interest and the nonneural artifacts that relate to the gait cycle since the interstimulus interval was 1,000 ms + random steps of 250 ms (interstimulus intervals were between 1000 and 2500 ms). This approach of analyzing a large number of specific ERPs promises that random noises associated with gait will not accumulate and signal-to-noise ratio (SNR) will be relatively high allowing quantifying the effects of motor cognitive functions. The 4 min resting state and the 4 min simple walking were not divided into epochs. EEG traces were visually reviewed by an experienced electroencephalographer, and the total duration (in seconds) of interictal epileptiform activity was annotated.

### Event-Related Potentials (ERPs)

**Figure 1:**
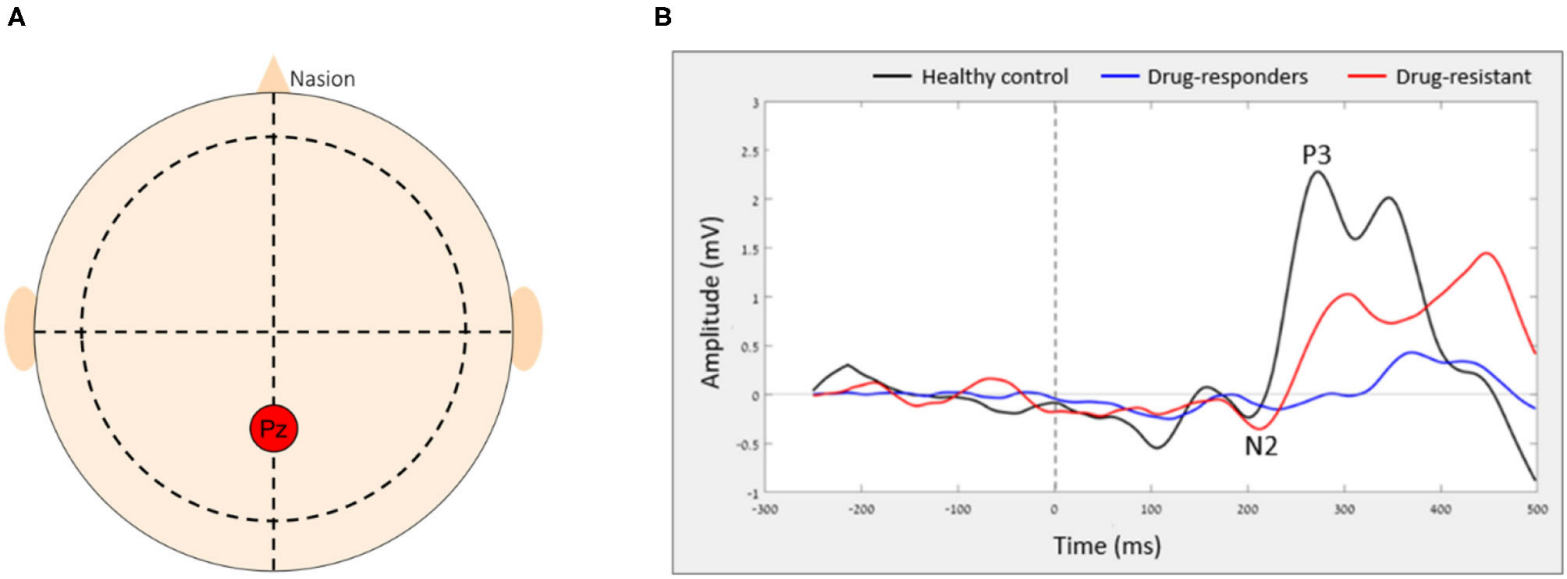
The average ERP of each group in Pz electrode. **(A)** The relative location of Pz electrode on the scalp and **(B)** the average ERP of each group.

ERP were isolated using averaging across multiple epochs. In this study, we examined the Go and NoGo ERPs, generated in the Pz electrode (Figure 1A) as it considered the location of maximal P3 wave (35–37) and previous studies showed differences in both P3 and N2 components between people with epilepsy and controls (38, 39). The NoGo N2 reflects a frontal inhibition mechanism that is active during NoGo trials (20, 40), while the NoGo P3 relates to inhibition (41, 42) or reset of a preceding inhibition process (15). After the ERPs were extracted, the amplitude and latency of N2 and P3 components were calculated. N2 peak amplitude [as defined by (37)] was defined as the most negative amplitude, surrounded on both sides by higher voltages, in the time window of 150–400 ms. The P3 peak amplitude was the highest amplitude occurring between 300 and 650 ms which was surrounded on both sides by lower voltages. For each peak amplitude (N2 and P3), a mean peak was calculated by averaging all amplitudes in a time window of 50 ms centered around the local peak to eliminate the evaluation of randomly high or low peak amplitude and minimize sensitivity to noise and artifacts (37). The latency was defined as the peak time point (poststimulus) in ms (Figure 1B). The dual-task effect on ERPs amplitude and latency was defined as the difference between walking and sitting (e.g., Walk P3 amplitude–Sit P3 amplitude = DT cost P3 amplitude). We also examined the ERPs from the Fz electrode that showed similar patterns of changes as the Pz electrode but to a lesser extent that did not reach significance. Therefore, we decided to include in this study only the results from the Pz electrode.

### Statistical Analysis

The mean and SD of all the demographic and cognitive variables were calculated and evaluated for normality and homogeneity of variance using the Q-Q plot and Levene's homogeneity test, respectively. One-way ANOVA and least significant difference (LSD) *post-hoc* analyses were used to examine differences between groups in demographics and behavioral measures. Gender differences were examined using the chi-squared test. An independent *t*-test was used to examine differences in epilepsy characteristics, which were tested only between the two JME groups. Differences in interictal activity (number and duration of interictal episodes) were tested using the Mann–Whitney *U* test due to abnormal distribution. All other variables were normally distributed. Linear mixed models were used to examine the effects of group (controls, drug-responsive, and drug-resistant), condition (sit, walk), task (Go, NoGo), and their interactions on measures of vGNG performance (correct % and response time) and measures of ERPs (N2 and P3 amplitude and latency). In addition, differences in the dual-task effects on ERPs between healthy controls and all people with JME and between people with drug-responsive and drug-resistant JM were examined using independent *t*-tests. Pearson's correlations between behavioral measures (correct % and response time, MoCA, and CTT) and ERPs measures (N2 and P3 amplitudes and latencies) were examined in all subjects together due to the small sample of our cohort. The significance level was set to *p* = 0.05 and corrected for multiple comparisons. The statistical analyses were performed using SPSS software.

## Results

### Participant Characteristics

Characteristics of participants are summarized in Table 1. Age and gender were similar across the groups (*F* = 1.974, *p* = 0.157, and χ^2^ = 0.634, *p* = 0.728). The comparison between healthy controls and all people with JME (drug-responsive and drug-resistant together) revealed a higher number of years of education (*t* = 3.203, *p* = 0.004, *d* = 1.350) and MoCA scores (*t* = 3.177, *p* = 0.004, *d* = 1.204) in healthy controls. Comparison of CTT scores between each patient group and healthy controls revealed that there was no difference between healthy controls and drug-responsive CTT scores (*t* = −1.279, *p* = 0.218, *d* = 0.556) but that healthy controls had higher scores than people with drug-resistant JME (W = 10, p = 0.006, Rank96 Biserial correlation = 0.750). In addition, people with drug-responsive JME had higher MoCA scores than drug-resistant (*t* = 2.113, *p* = 0.050, *d* = 0.982).

**Table 1:** Subject characteristics, significant values are marked in yellow.

**Variable**	**Healthy** **(*n* = 13)**	**JME** **(*n* = 19)**	***p* values**	**Drug-responsive (*n* = 11)**	**Drug-resistant** **(*n* = 8)**	***p* values**
**Demographic**						
Age (years)	29.46 ± 0.69	29.05 ± 1.35	0.816	31.50 ± 1.50	27.27 ± 2.30	0.126
Gender (M/F)	7/6	7/12	0.357	4/7	3/5	0.962
Education (years)	17.88 ± 0.88	14.39 ± 0.60	0.004	15.00 ± 0.88	13.56 ± 0.70^**^	0.248
**Epilepsy characteristics**						
Epilepsy duration (years)	-	-	-	9.63 ± 1.92	15.37 ± 3.12	0.118
last month seizures number	-	-	-	1.72 ± 1.26	5.37 ± 4.95	0.422
num of current med	-	-	-	1.81 ± 0.26	3.00 ± 0.46	0.030
**Cognitive**						
MoCA	28.46 ± 0.58	25.05 ± 0.74	0.004	26.27 ± 0.59	23.38 ± 1.40^**^	0.050
CTT (B-A) (sec)	26.73 ± 5.15	52.03 ± 9.17	0.067	38.63 ± 7.57	70.46 ± 17.81^**^	0.086
**vGNG - Sitting (single task)**						
Correct press (%)	99.64 ± 1.56	94.96 ± 2.01	0.003	94.12 ± 3.29	96.16 ± 0.12	0.634
Correct avoid (%)	87.69 ± 4.97	73.82 ± 3.47	0.004	74.75 ± 4.961^*^	72.50 ± 1.92^**^	0.761
Total correct (%)	97.25 ± 1.54	90.73 ± 1.74	<0.001	90.25 ± 2.83	91.42 ± 0.41	0.751
Reaction time (ms)	355.4 ± 11.2	378.9 ± 13.5	0.238	365.4 ± 11.0	398.2 ± 21.4	0.247
**vGNG - Walking (dual-task)**						
Correct press (%)	98.82 ± 4.64	94.69 ± 2.03	0.131	96.27 ± 1.62	92.21 ± 0.48	0.344
Correct avoid (%)	84.16 ± 6.78	71.44 ± 4.46	0.075	70.11 ± 6.11	73.54 ± 3.12	0.719
Total correct (%)	95.89 ± 3.48	90.05 ± 1.93	0.029	91.04 ± 2.35	88.48 ± 0.76	0.535
Reaction time (ms)	368.5 ± 11.8	382.6 ± 12.5	0.440	360.3 ± 16.2	423.7 ± 22.7^**^	0.010

* *Significant difference between healthy and drug-responsive*.

** *Significant difference between healthy and drug-resistant*.

Between the two patient groups, there were no differences in epilepsy duration (*t* = −1.647, *p* = 0.118, *d* = −0.765) and the number of seizures in the previous month (*t* = −0.822, *p* = 0.422, *d* = −0.519). People with drug-resistant JME were prescribed a higher number of current medications (*t* = −2.367, *p* = 0.030, *d* = −1.100). Interictal activity was detected in four people with JME, two drug-responsive and two drug-resistant. No differences in the number of interictal episodes (*W* = 103.5, *p* = 0.360) and their duration (*W* = 103.5, *p* = 0.360) were found between the two patient groups. In addition, no differences in the interictal activity during sitting and walking were found (number of episodes: *W* = 143.5, *p* = 0.406 and episodes duration: *W* = 143.0, *p* = 0.422).

### Visual Go/NoGo

**Figure 2:**
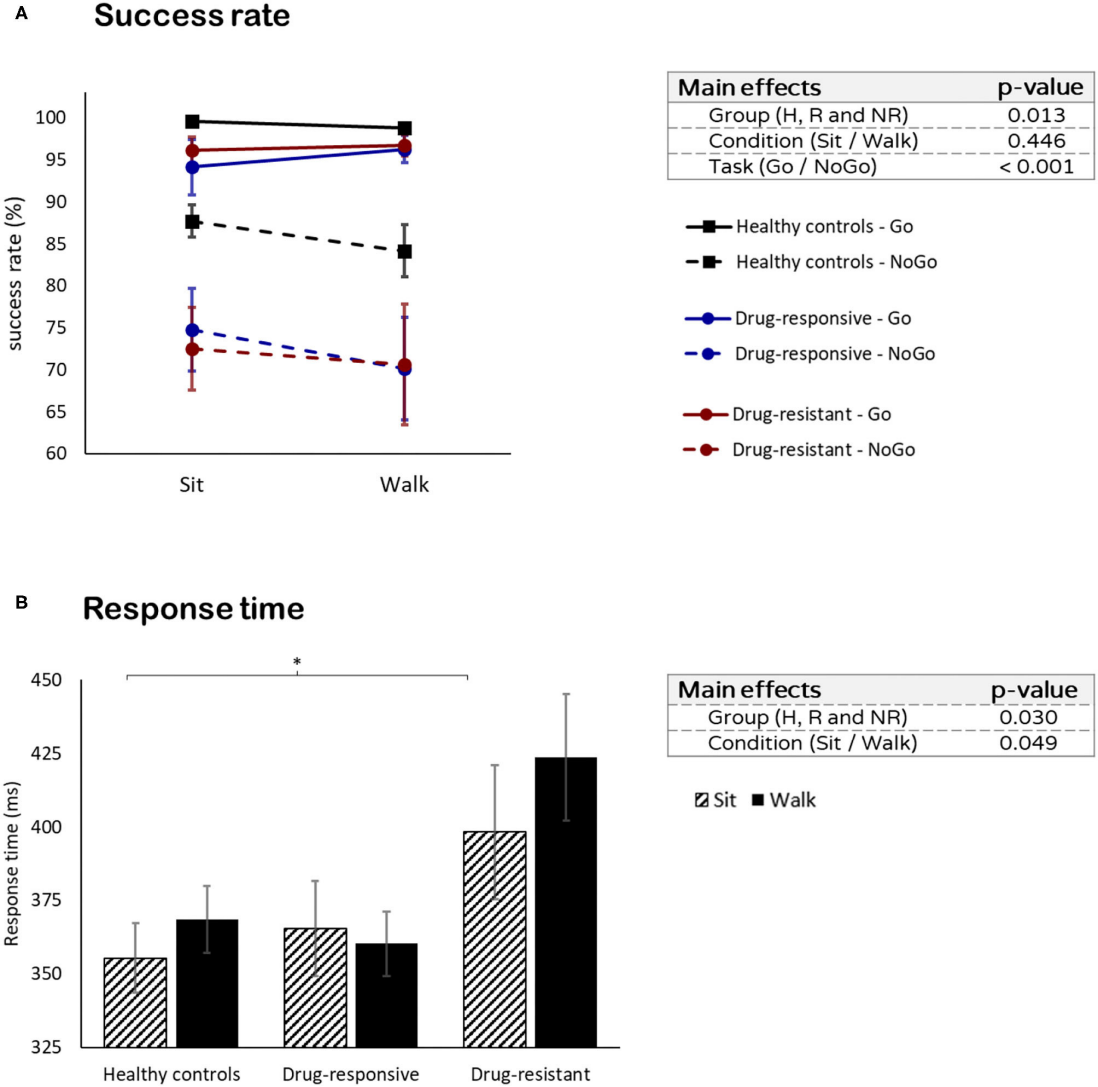
Visual Go/NoGo task performance: **(A)** success rate in the Go (solid line) and NoGo (dashed line) tasks and **(B)** the response times while sitting and while walking. ^*^p ≤ 0.05.

Healthy controls had a higher success rate than both drug-responsive (*t* = 3.890, *p* < 0.001) and drug-resistant (*t* = 3.295, *p* = 0.004) (group effect: *F* = 5.032, *p* = 0.013, η^2^*p* = 0.161). No differences in the success rate between sitting and walking were found in all groups (condition effect: *F* = 0.435, *p* = 0.511, η^2^*p* = 0.000) (Figure 2A). However, a comparison between Go and NoGo trials revealed that the success rate was higher in the Go trials than in the NoGO trials in all groups (task effect: *F* = 68.964, *p* < 0.001, η^2^*p* = 0.484). There was no difference in success rates between drug-responsive and drug-resistant (% total success when sitting: *t* = −0.323, *p* = 0.751, and % total success when walking: *t* = 0.635, *p* = 0.535).

No differences in reaction time were found between healthy controls and all people with JME while sitting (*t* = −1.209, *p* = 0.238) and while walking (*t* = −0.784, *p* = 0.440). However, people with drug-resistant JME had a longer response time than drug-responsive (*t* = −3.006, *p* = 0.011) and healthy controls (*t* = −3.090, *p* = 0.009) (group effect: *F* = 4.011, *p* = 0.030, η^2^*p* = 0.186). No difference in response time between sitting and walking was observed in all groups (condition effect: *F* = 0.799, *p* = 0.376, η^2^*p* = 0.016) (Figure 2B).

### ERPs Amplitude and Latency

#### P3 Amplitude

**Figure 3:**
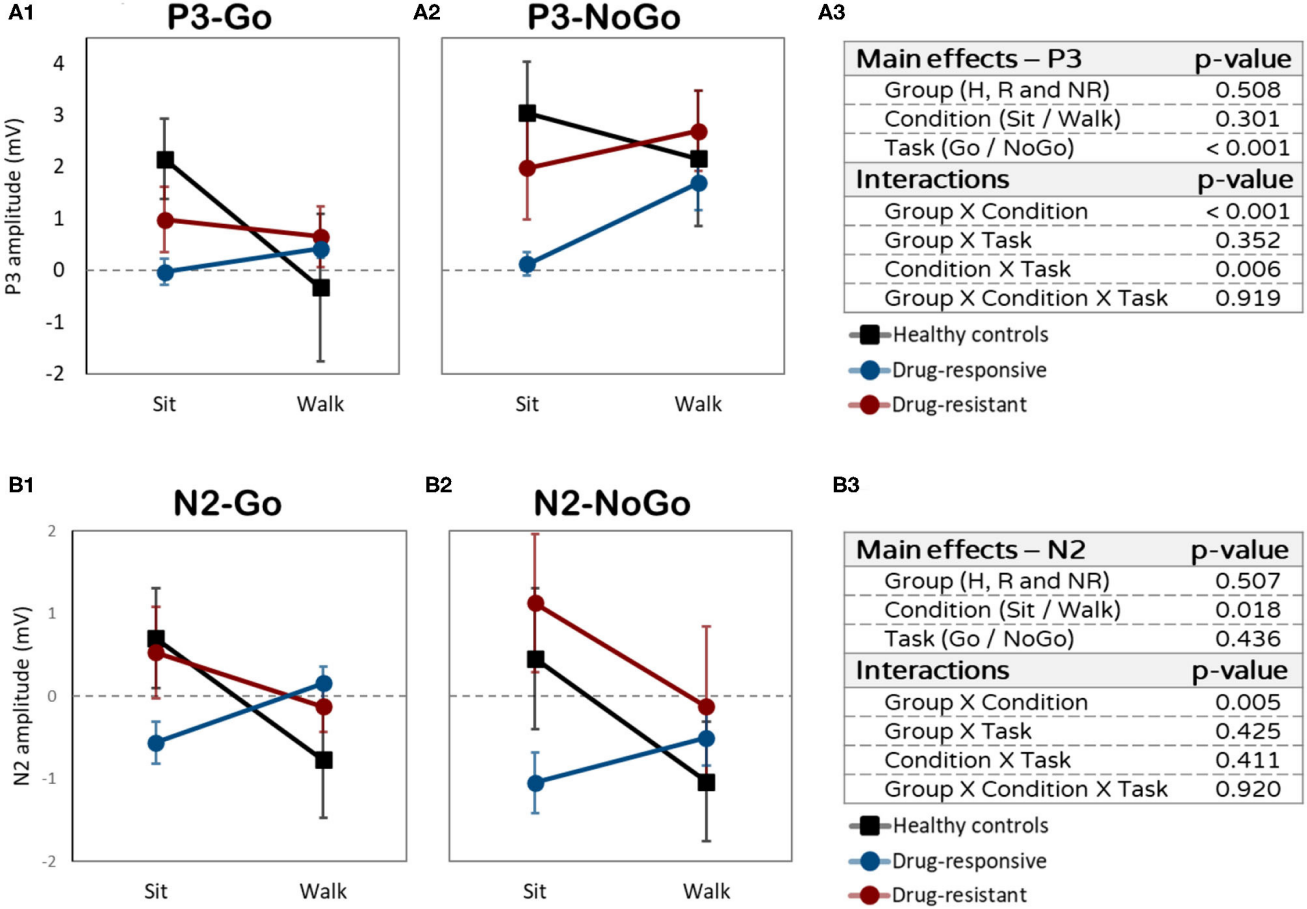
Amplitudes differences (in mV) between sitting and walking. **(A1)** P3 amplitudes while performing the Go task. **(A2)** P3 mplitudes while performing the NoGo task. **(A3)** P3 effects and interactions summery. **(B1)** N2 amplitudes while performing the Go task. **(B2)** N2 amplitudes while performing the NoGo task. **(B3)** N2 effects and interactions summary.

Although there was no main effect of condition (sitting vs. walking, *F* = 1.115, *p* = 0.301, η^2^*p* = 0.001), interactions were found between task (Go or NoGo) and condition and between group and condition. All 3 groups showed a higher P3 amplitude for NoGo than for Go during walking but not during sitting (interaction task X condition: *F* = 8.818, *p* = 0.006, η^2^*p* = 0.016). While performing the Go task, the healthy controls had a higher P3 amplitude during sitting than during walking (*t* = 3.175, *p* = 0.013) (Figure 3A). People with drug-responsive JME had a higher P3 amplitude during walking than during sitting, while performing the NoGo task (*t* = −2.387, *p* = 0.041) (Figure 3B). People with drug-resistant JME did not show a difference in P3 amplitude between sitting and walking (interaction group X condition: *F* = 9.817, *p* < 0.001, η^2^*p* = 0.054).

#### N2 Amplitude

A main effect of condition (sitting vs. walking: *F* = 6.369, *p* = 0.018, η^2^*p* = 0.025) and interaction effect between group and condition (*F* = 6.597, *p* = 0.005, η^2^*p* = 0.061) were found for N2 amplitude. Healthy controls and drug-resistant had more prominent (more negative) N2 amplitudes during walking than during sitting, while performing the Go and NoGo tasks (Figure 3B). Drug-responsive demonstrated the opposite pattern, less prominent N2 amplitude during walking compared to sitting. The dual-task effect on N2 amplitude was significantly different between drug-responsive and drug-resistant (*t* = 2.120, *p* = 0.050), drug-responsive showing a larger dual-task effect than drug-resistance.

#### P3 Latency

**Figure 4:**
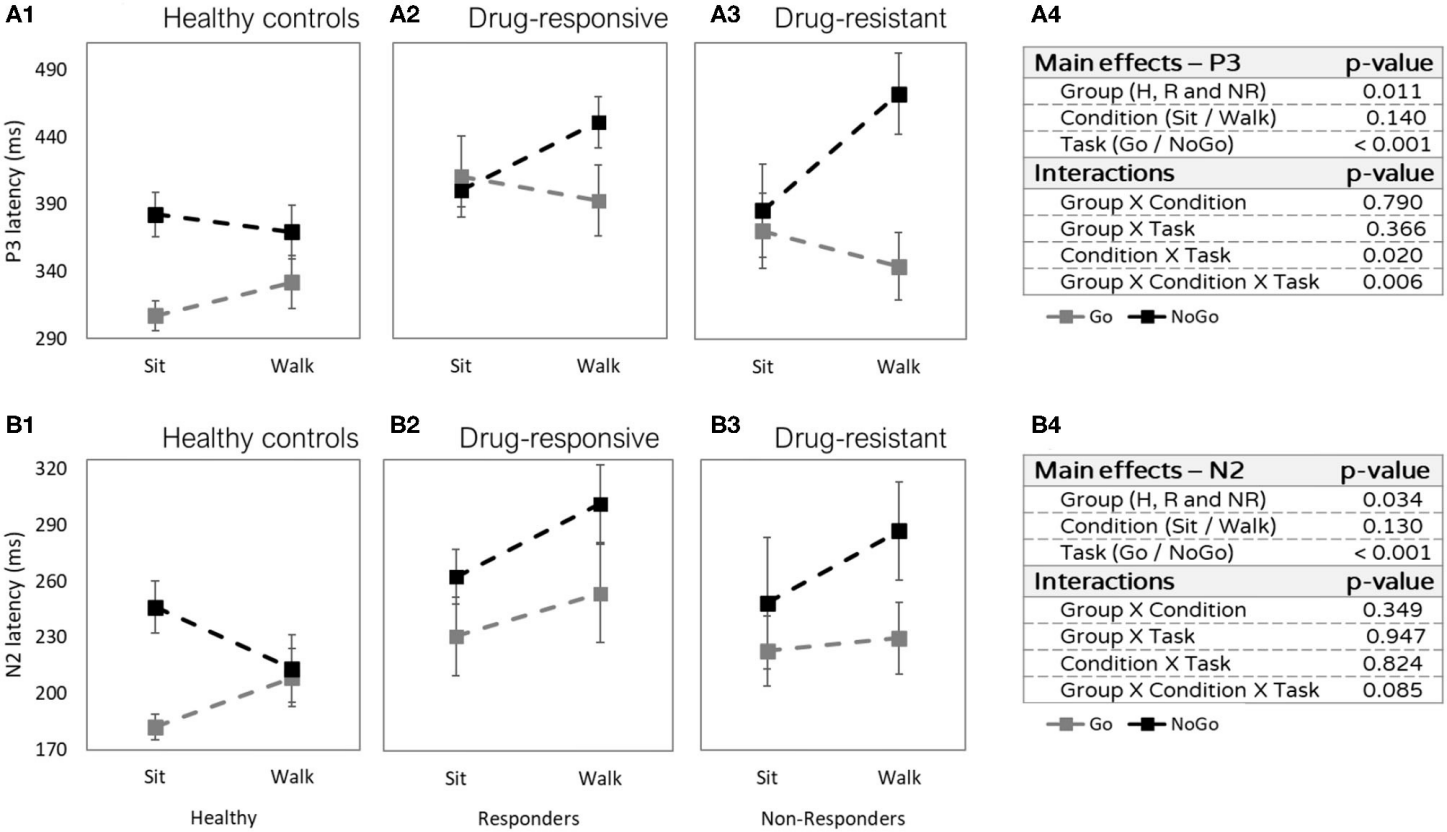
Latencies differences (in milliseconds) between sitting and walking (Y axis), in the Go task (gray) and in the NoGo task (black). **(A1)** P3 latencies of healthy controls. **(A2)** P3 latencies of people with drug-responsive JME. **(A3)** P3 latencies of people with drug-resistant JME. **(A4)** P3 effects and interactions summery. **(B1)** N2 latencies of healthy controls. **(B2)** N2 latencies of people with drug-responsive JME. **(B3)** N2 latencies of people with drug-resistant JME. **(B4)** N2 effects and interactions summary.

No main effect for condition (sitting vs. walking: *F* = 2.313, *p* =4 0.140, η^2^*p* = 0.016) was observed. However, we found interactions between task and condition and between group, task, and condition. The P3 latency was shorter in the Go task compared to the NoGo task (task effect: *F* = 15.984, *p* < 0.001, η^2^*p* = 0.122) and this difference was more prominent while walking than while sitting (interaction task × condition *F* = 6.171, *p* = 0.020, η^2^*p* = 0.030). Specifically, the difference was more prominent during walking in the two patient groups (Figures 4A2,A3), but not in the healthy control group (Figure 4A1) which showed earlier Go latency while walking and while sitting (interaction group × task × condition: *F* = 6.360, *p* = 0.006, η^2^*p* = 0.052).

#### N2 Latency

The N2 latency was shorter in healthy controls compared to both patient groups (main effect group: *F* = 3.814, *p* = 0.034, η^2^*p* = 0.117, *post hoc* healthy controls vs. drug-responsive: *t* = −3.675, *p* = 0.001, and healthy controls vs. drug-resistant: *t* = −2.385, *p* = 0.049). In addition, N2 latency was shorter in the Go task compared to the NoGo task (main effect task: *F* = 17.177, *p* < 0.001, η^2^*p* = 0.094) (Figure 4B). The difference in N2 latencies between sitting and walking was not significant (*F* = 2.440, *p* = 0.130, η^2^*p* = 0.019).

### Correlations

**Figure 5:**
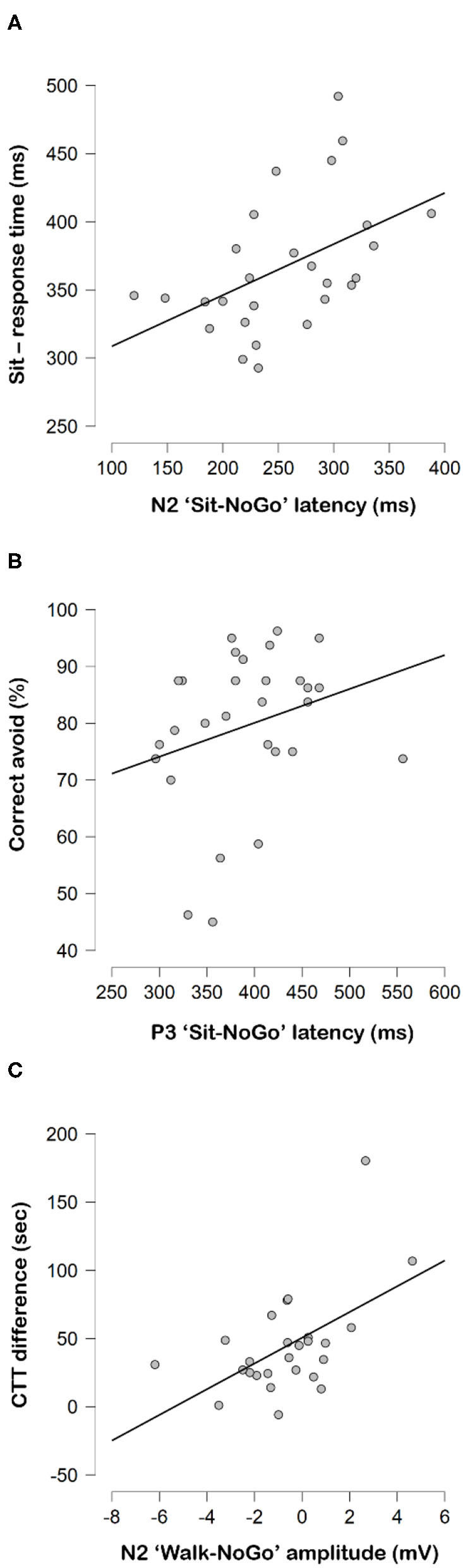
Correlations between **(A)** N2 “Sit-NoGo” latencies to response time while sitting, **(B)** P3 “Sit-NoGo” latencies to correct avoid rates (%) and **(C)** CTT difference and N2 “Walk-NoGo.”

Longer “Sit-NoGo” N2 latencies correlated to longer reaction times while sitting (*r* = 0.457, *p* = 0.019) (Figure 5A), but longer “Sit-NoGo” P3 latencies correlated to higher correct avoidance (NoGo) rate while sitting (*r* = 0.411, *p* = 0.030) (Figure 5B). More pronounced (more negative) N2 “Walk-NoGo” amplitudes were correlated to better CTT scores (*r* = 0.510, *p* = 0.011) (Figure 5C).

## Discussion

This study explored the differences in electrical brain activity during a vGNG task performed while seated and while walking on a treadmill, between people with drug-responsive and drug-resistant JME. Our findings showed delayed N2 and P3 latencies mainly during walking in people with JME compared to healthy controls. Comparison between drug-responsive and drug-resistant revealed different dual-task effects on N2 amplitude, in the drug-responsive group, N2 was less prominent with walking while in the drug-resistant group it was more prominent with walking. This difference in N2 amplitude was accompanied by a slower reaction time during dual-task walking in the drug-resistant group. These alterations may shed light on the underlying neural mechanism that contributes to attentional deficits in people with JME.

### Brain Activity

#### N2 and P3 Amplitudes and Latencies While Sitting

The only difference found between the two patient groups was more prominent N2 amplitude in the drug-responsive compared to drug-resistant. This difference was more pronounced in the NoGo task than in the Go. N2 is one of the early components of ERP associated with orienting attention to relevant stimuli (43). The less prominent N2 amplitude found in drug-resistant, together with their worse performance, may reflect alterations in the early processing of the task stimuli (44–46). The correlation between less prominent NoGo N2 amplitude and worse CTT performance further supports these deficits in attention and control inhibition in people with JME (30). The less prominent N2 amplitude during the NoGo task in people with drug-resistant JME, along with their lower vGNG task's success rates, may reflect the difficulty in allocating neuronal resources (44–46). Very little research has been done on the effects of ASMs on the N2 amplitudes of people with JME, or people with epilepsy in general. Further research should examine whether less prominent N2 amplitude characterizes naive to drugs patients and whether it can predict the responsiveness status.

In line with Soysal et al. (27), we did not find differences in ERPs latencies between the two patient groups. However, in contrast to several studies, which did not show a significant change in the latencies of P3 and N2 in people with generalized epilepsy compared to healthy controls (24, 25, 27), we found significant differences between healthy controls and people with JME. Healthy controls had earlier N2 and P3 latencies compared to people with JME, mainly in the “Walk-NoGo” condition. Previous studies have found shorter P3 latencies after ASMs treatment (47), and some drugs have shown a greater effect than others, for example, Levetiracetam had a greater effect than Carbamazepine, and Sodium Valproate. However, despite the higher number of ASMs used by the drug-resistant subjects, we did not find differences in N2 and P3 latencies between the two JME groups. A larger sample of people with epilepsy is needed to further explore the effects of drugs on ERPs amplitude and latencies.

#### Dual-Task Effects on N2 and P3 Amplitudes and Latencies

People with JME showed different patterns of change in NoGo N2 and P3 latencies during sitting and walking (dual-task effect) compared to healthy controls (Figure 4). While people with JME demonstrated longer latencies during walking compared to sitting, healthy controls presented the opposite, shorter latencies during walking compared to sitting. The longer latencies during sitting, which was considered the easier task, in healthy controls may suggest that they were less engaged in the vGNG task during sitting. On the other hand, performing the task during walking was more challenging for them and therefore required a higher level of engagement that improved cognitive processing and reduced latency. An additional explanation is that walking requires greater activation in brain areas that increase arousal and as a result facilitates the engagement in the secondary cognitive task. Unlike the healthy controls, people with JME found the task difficult already during sitting and this difficulty further increased during walking. These interpretations should be taken with caution, as we did not debrief the participants after completing the tasks.

As for the ERPs amplitude, each group demonstrated different patterns of change in N2 and P3 amplitudes during sitting and walking (dual-task effect). It is important to note that since P3 reflects a positive wave, reduced amplitude manifests a lower electrical response (37). In contrast, N2 reflects a negative wave, therefore, reduced amplitude manifests a higher electrical response. The fact that healthy controls had lower Go and NoGo P3 amplitude while walking is not surprising and is consistent with existing knowledge (16). Walking requires multiple brain resources, which reduces neural synchronization and availability of cognitive resources, both leading to lower P3 amplitude [(17, 48, 49)]. In contrast, both JME groups tended to increase P3 amplitude during walking compared to sitting, suggesting that people with JME rely more on attentional resources associated with P3 during walking and less on other resources associated with the motor system. However, a different pattern of dual-task effect was found in N2 amplitude. While healthy controls and people with drug-resistant JME showed more prominent N2 (more negative) during walking compared to sitting, people with drug-responsive JME demonstrated the opposite, less prominent N2 amplitude during walking compared to sitting. These findings may suggest the recruitment of additional brain resources in the attentional preprocessing stage in people with drug-responsive JME that helped them to maintain high motor and cognitive performance (50). On the other hand, people with drug-resistant JME were not able to change the pattern of activation during the more demanding task, which may explain their worse vGNG performance during walking.

### Cognitive Performance

Many studies have shown deficits in response inhibition in people with JME (13), indicating cognitive impulsivity that directly affects decision-making [(51–54)]. In line with these studies, our results demonstrated that both people with drug-resistant and drug-responsive JME had more commission errors (NoGo errors) than the healthy controls. However, only drug-resistant demonstrated a longer response time in the Go task and worse performance in the CTT and MoCA tests compared to healthy controls. These findings indicate that people with drug-resistant JME have more severe cognitive impairments that encompass additional cognitive abilities, such as attention, processing speed, and cognitive flexibility.

In contrast to our study, other studies that characterized the cognitive status of people with JME did not separate drug-responsive and drug-resistant. Given the prevalence of drug-resistant, it is likely that they accounted for up to 30% of the study sample which means that most of the information available in the literature pertains mainly to drug-responsive. In our study, the drug-resistant accounted for 42% of the people with JME, as our main goal was to compare between drug-responsive and drug-resistant. Therefore, it is possible that by combining the two groups, we attributed lower cognitive abilities to drug-responsive that masked the real differences between these groups and healthy controls. Drug-resistant people presented lower MoCA scores and longer response times in the Go task while dual-tasking, compared to people with drug-responsive JME and healthy controls, indicating that their global cognition and processing speed are significantly impaired.

### Limitations

There are several limitations we would like to recognize. First, the size of our sample was relatively small, larger samples of drug-responsive and drug-resistant may yield more significant results relative to the differences between the two groups. Due to the small number of subjects and the desire to admit as many people with JME as possible, we chose not to disqualify according to the types and doses of the ASMs, which resulted in a wide variety of medications taken by the subjects. In addition, the patients took their medications at their regular hours and were tested at different times of the day. Some subjects were tested immediately after taking the medications and some subjects several hours later. The different medications and time of examinations could have had effects on brain activity and cognition; therefore, follow-up studies should be conducted on both naive people with epilepsy and populations taking specific ASMs. The two groups of people with epilepsy in our study did not differ from each other in the duration of the disease, nor in the number of seizures in the past month, nor the number of interictal events. These are all measures that we expected to be different between the two groups. Nevertheless, the directionality of the data was in line with the hypotheses, and we assume that a larger sample would have been able to yield significant differences. Another limitation worth noting is that walking on a treadmill while holding the trails is less demanding than walking over ground and therefore, might have reduced higher interference of dual-task on neural control.

### Conclusions

This study demonstrates the feasibility of measuring ERP metrics (latencies and amplitudes of N2 and P3) during a dual task that combines vGNG and walking, as a tool to study abnormal functional networks in people with epilepsy. Our findings suggest that more prominent N2 amplitude during walking in the early attentional preprocessing stage and worse cognitive performance may be used as a potential marker to distinguish between people with drug-resistant JME and people with drug-responsive JME. The main limitations of our study are the small sample size and the various medications used by the subjects. Thus, our results emphasize the need for future studies that include a larger sample size and drug-naïve patients.

## Data Availability Statement

The raw data supporting the conclusions of this article will be made available by the authors, without undue reservation.

## Ethics Statement

The studies involving human participants were reviewed and approved by Ministry of Health of Israel MOH_2018-10-31_004724. The patients/participants provided their written informed consent to participate in this study.

## Author Contributions

MY collected the data, performed the analysis, and wrote the paper. SG collected the data and helped in data analysis. SN performed the calculations of measurements and helped in data analysis. AM, JH, LG, and NG aided in interpreting the results and worked on the manuscript. FF and IM conceived and designed the study, helped in data analysis, aided in interpreting the results, and worked on the manuscript. All authors contributed to the article and approved the submitted version.

## Funding

This work was supported in part by the Parasol Foundation.

## Conflict of Interest

The authors declare that the research was conducted in the absence of any commercial or financial relationships that could be construed as a potential conflict of interest. The handling editor declared a past co-authorship with several of the authors AM and IM.

## Publisher's Note

All claims expressed in this article are solely those of the authors and do not necessarily represent those of their affiliated organizations, or those of the publisher, the editors and the reviewers. Any product that may be evaluated in this article, or claim that may be made by its manufacturer, is not guaranteed or endorsed by the publisher.
